# Die Rolle des entzündlichen Zelltods bei Typ‐1‐dominanten entzündlichen Hautkrankheiten

**DOI:** 10.1111/ddg.70066

**Published:** 2026-08-04

**Authors:** Felix Lauffer

**Affiliations:** ^1^ Klinik für Dermatologie und Allergologie LMU Klinikum Ludwig‐Maximilians‐Universität München München Deutschland

**Keywords:** Apoptose, Interface Dermatitis, Nekroptose, Zelltod, apoptosis, cell death, Interface Dermatitis, necroptosis

## Abstract

Während es in den letzten Jahren große Fortschritte bei der Entwicklung neuer Therapien für die Psoriasis und die atopische Dermatitis gab, ist die Innovation im Bereich der Typ‐1‐dominanten Hautkrankheiten immer noch überschaubar. Zu dieser Gruppe zählt man all jene Erkrankungen, bei denen es zu einer zytotoxischen Immunreaktion gegen residente Zellen der Haut kommt. Das histologische Pendant ist eine Interface‐Dermatitis, also ein subepidermal gelegenes entzündliches Infiltrat begleitet von epidermalem Zelltod. Zu dieser Gruppe zählt man unter anderem den Lichen planus, den kutanen Lupus erythematodes, das Erythema exsudativum multiforme, die Alopecia areata und die Vitiligo. Immunologisch zeigt sich eine Dominanz von Th1‐Zellen, welche ihre Effekte durch Interferon‐γ und Tumornekrosefaktor‐α vermitteln. Neuere Erkenntnisse haben gezeigt, dass neben der Apoptose auch weitere Zelltodformen durch diese Immunreaktion aktiviert werden, wie zum Beispiel die Nekroptose. Im Gegensatz zur Apoptose stellt die Nekroptose einen starken immunologischen Stimulus dar und verstärkt so noch einmal die lokale Entzündungsreaktion. Dies eröffnet neue therapeutische Möglichkeiten, da aktuell zahlreiche Nekroptose‐Inhibitoren für verschiedene entzündliche Erkrankungen untersucht werden. Dieser Übersichtartikel fasst die Immunpathogenese der Typ‐1‐dominanten Hauterkrankungen zusammen und beleuchtet neue therapeutische Optionen, wie die Inhibition des entzündlichen Zelltods.

## DAS KLINISCHE PROBLEM

**ABBILDUNG 1:**
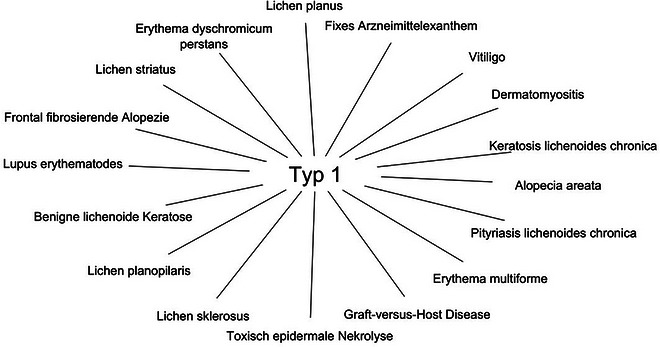
Übersicht über die Erkrankungen, die zur Gruppe der Typ‐1‐dominanten entzündlichen Hauterkrankungen gezählt werden.

Entzündliche Dermatosen betreffen bis zu 20% der Bevölkerung in westlichen Ländern. Während es in den letzten Jahren große Fortschritte bei der Psoriasis, der atopischen Dermatitis und der Hidradenitis suppurativa gab,^1^, ^2^ ist die Entwicklung neuer therapeutischer Ansätze für Typ‐1‐dominante entzündliche Hauterkrankungen bisher noch nicht sehr weit fortgeschritten. Unter diesen versteht man all jene Hauterkrankungen, bei denen es zu einer zytotoxischen Immunreaktion gegen residente Zellen der Haut kommt. Histologisch kann man diese als Interface‐Dermatitis nachvollziehen, welche definiert ist als das Zusammenkommen eines subepidermal gelegenen entzündlichen Infiltrates, vorwiegend aus Lymphozyten bestehend, und Zelltod innerhalb der Epidermis.^3^ Immunologisch wird diese Entzündungsreaktion durch T‐Helfer‐Zellen 1 (Th1‐Zellen) und zytotoxische T‐Zellen vermittelt,^4^ was zu der Bezeichnung Typ‐1‐dominante entzündliche Hauterkrankungen führte.^5^ Auch wenn jede einzelne Erkrankung eine spezifische Immunpathogenese aufweist, werden nach dieser Definition alle Erkrankungen zu einer Gruppe zusammengefasst, die histologisch entweder eine Interface‐Dermatitis zeigen oder bei denen andere residente Zellen der Haut immunologisch attackiert werden, was zum Zelltod führt.^6^ Hieraus resultiert, dass auch die Vitiligo, bei der sich eine zytotoxische Immunantwort gegen Melanozyten richtet oder die Alopecia areata, bei der der Haarfollikel Zielorgan der Immunreaktion ist, zur Gruppe der Typ‐1‐dominanten entzündlichen Hauterkrankungen gezählt werden. Weitere Erkrankungen dieser Gruppe sind in Abbildung 1 aufgelistet. Physiologisch wird die Typ‐1‐Immunität benötigt, um Viren zu eliminieren oder entartete Zellen zu beseitigen.^7^ Es handelt sich hierbei also um eine Reaktion auf intrazellulär gelegen Prozesse, bei denen das Immunsystem keinen Erreger phagozytieren oder mit Antikörpern binden kann, um ihn zu eliminieren. Die Schlüssel Zytokine Interferon (IFN)‐γ und Tumornekrosefaktor (TNF)‐α stimulieren die Aktivität zytotoxischer Zellen und vermitteln über verschiedene Rezeptoren auch direkt Zelltod. Die aktuellen Therapiemöglichkeiten für Typ‐1‐dominante Hauterkrankungen beschränken sich weitestgehend auf Kortikosteroide, Retinoide, UV‐Therapie und breit wirksame Immunsuppressiva. Es gibt daher einen hohen Bedarf, die Pathomechanismen dieser Erkrankungsgruppe besser zu verstehen, um neue therapeutische Ansatzpunkte zu definieren.

## HYPOTHESE

Unsere primäre Hypothese ist, dass der epidermale Zelltod zur Entzündungsreaktion bei Typ‐1‐dominanten Hautkrankheiten beiträgt und somit einen potenziell neuen therapeutischen Ansatzpunkt darstellt. Unsere Forschung zielt also darauf ab, die Mechanismen des epidermalen Zelltodes genauer zu verstehen und spezifische Inhibitoren auf ihre potenziell antientzündlichen Effekte bei dieser Erkrankungsgruppe zu untersuchen.

## FORSCHEN FÜR DIE PRAXIS

### Übersicht über die Formen des Zelltodes

Es gibt verschiedene Mechanismen, die dazu führen, dass eine Körperzelle abstirbt (Tabelle 1). Die unregulierte Form ist die Nekrose, bei der eine Zelle aufgrund von Unterversorgung oder physikalischen Einflüssen (Trauma, Verätzung, Verbrühung etc.) nicht ihre physiologische Funktion aufrechterhalten kann. Im Gegensatz dazu laufen die Apoptose, die Nekroptose, die Ferroptose und die Pyroptose in bestimmten Schritten ab, die bis zu einem gewissen Punkt reversibel sind. Die Apoptose trägt zur physiologischen Regeneration von Zellen, Geweben und Organen bei und löst keine weitere Entzündungsreaktion aus. Sie kann sowohl intrinsisch als auch extrinsisch ausgelöst werden und wird durch Caspasen gesteuert. Extrazellulär kann die Bindung an Todesrezeptoren die Apoptose auslösen. Intrinsisch läuft die Regulation über die Freisetzung von Cytochrom C aus den Mitochondrien. Beide Wege resultieren in einer Degradierung der DNA und einer Zersetzung der Zelle in kleinere Vesikel, die dann von phagozytierenden Zellen abgebaut werden. Da jedoch verschiedenen Viren sich es zu Nutze gemacht haben, die Apoptose gezielt zu hemmen und so einen Vermehrungsvorteil zu erlangen,^7^ bedarf es weiterer Mechanismen des Zelltodes, die unabhängig von der Apoptose sind. Die Nekroptose wird durch einen Komplex von RIPK1 und RIPK3 reguliert, die gemeinsam das Nekrosom bilden. Dieser aktiviert MLKL, welches sich mit weiteren Molekülen zusammenlagert und Poren in der äußeren Zellmembran bildet. Es kommt hierdurch zu einem unkontrollierten Einstrom von Flüssigkeit in die Zelle, wodurch die Zelle anschwillt und platzt. Die freigesetzten Zellorganellen, sowie die DNA stellen für Immunzellen einen starken Stimulus dar, da sie an Musterkennungsrezeptoren binden und so eine Entzündungsreaktion antreiben.^8^ Ein weiterer entzündlicher Zelltod ist die Pyroptose. Sie wird durch eine Aktivierung des Inflammasoms ausgelöst und führt ebenfalls zur Bildung von Poren an der äußeren Zellmembran, welche durch Proteine der Gasdermin‐Familie gebildet werden. Wie bei der Nekroptose schwillt die Zelle an und platzt. Zusätzlich werden Interleukin (IL)‐1β und IL‐18 ausgeschüttet, welche eine starke Entzündungsreaktion induzieren.^9^ Zuletzt ist die Ferroptose zu nennen, bei der es durch freie Sauerstoffradikale zu einer Lipidoxidierung und zu einem Membranschaden der Zelle kommt. Namensgebend für die Ferroptose ist die Anreichung von Eisen in der Zelle, die die Generation freier Sauerstoffradikale fördert.^10^ Es gibt in der Literatur auch Berichte über die gleichzeitige Aktivierung mehrerer Zelltodformen, die sogenannte PANoptose.^11^ Sie wurde insbesondere im Zusammenhang mit schweren viralen Infektionen, wie zum Beispiel der SARS‐COV2‐Pneumonie, beschrieben.^12^ Interessanterweise wurden spezifische Inhibitoren des entzündlichen Zelltodes auch in dieser Population getestet. So zeigte eine Phase‐1b‐Studie des RIPK1‐Inhibitors Eclitasertib bei Patienten mit einer schweren COVID‐19‐Erkrankung eine tendenziell schnellere Verbesserung inflammatorischer Biomarker und klinischer Symptome in der Verum‐Gruppe im Vergleich zum Placebo, bei insgesamt guter Verträglichkeit.^13^ Weitere Moleküle, die RIPK1, RIPK3 oder MLKL binden beziehungsweise hemmen, befinden sich in der Entwicklung für entzündliche Darmerkrankungen, rheumatologische Erkrankungen, Morbus Alzheimer, amyotrophe Lateralsklerose (ALS) und weitere Indikationen.^14^

**TABELLE 1:** Übersicht über die wichtigsten Charakteristika verschiedener Zelltodformen.

	Nekrose	Apoptose	Nekroptose	Pyroptose	Ferroptose
Aktivierung	Unzureichende Sauerstoff‐/Nährstoffversorgung Physikalische Noxen	*Extrinsisch*: Todesrezeptoren (z.B. FAS‐Ligand) *Intrinsisch*: Cytochrom‐C‐Freisetzung aus Mitochondrien	Tumornekrosefaktor‐α, Interferon‐γ, Toll‐Like‐Rezeptoren, Stress‐Signale	Toll‐Like‐Rezeptoren, NOD‐Like‐Rezeptoren, Inflammasom	Eisenoxidation, freie Sauerstoffradikale,
Wichtige Mediatoren	–	*Initiator‐Caspasen*: 8, 9 *Effektor‐Caspasen*: 3, 6, 7	ZBP1, TRIF, RIPK1, RIPK3, MLKL	Caspase 1	Proteinkinase A, GPX4
Mechanismus	Unkontrollierter Zelluntergang	Fragmentierung der Zelle	Poren in der Zellmembran, Platzen der Zelle	Poren in der Zellmembran, Platzen der Zelle	Schädigung der Zellmembran durch Lipidperoxidierung

### Entzündlicher Zelltod trägt zur Entzündungsreaktion bei Typ‐1‐dominanten entzündlichen Hautkrankheiten bei

Typ‐1‐dominante Hauterkrankungen zeichnen sich durch eine zytotoxische Immunreaktion aus, die sich histologisch als Interface‐Dermatitis präsentiert. Um die Mechanismen, die eine Interface‐Dermatitis steuern, näher zu untersuchen, haben wir Hautbiopsien von Patienten mit Lichen planus und kutanem Lupus erythematodes mithilfe von Transkriptom‐Untersuchungen analysiert. Hierbei zeigte sich eine starke Interferon‐vermittelte Immunreaktion sowie eine Aktivierung von Apoptose‐ und Nekroptose‐vermittelnden Genen. Weitere Versuche konnten zeigen, dass eine Hemmung von RIPK3, einem Schlüsselproteinen der Nekroptose, zu einer signifikanten Reduktion des Zelltodes von Keratinozyten führt, wenn diese mit INF‐γ und TNF‐α stimuliert wurden.^15^ Passend zu unseren Daten zeigte eine Studie einer anderen Forschungsgruppe, dass ZBP1, ein wichtiger Regulator der RIPK3‐abhängigen Nekroptose, bei der Graft‐versus‐Host‐Disease stark exprimiert ist. ZBP1 wird in humanen Keratinozyten durch IFN‐γ induziert und vermittelt unabhängig von RIPK1 die Induktion der Nekroptose.^16^ Auf der anderen Seite gibt es jedoch auch zelleigene Mechanismen, die Keratinozyten vor entzündlichem Zelltod schützen, wie zum Beispiel die *death‐associated protein kinase* 1 (DAPK1). Wir haben beobachtet, dass DAPK1 stark beim Lichen planus exprimiert wird und mit dem Schweregrad der Interface Reaktion korreliert. Der *Knock‐out* von DAPK1 in Keratinozyten führte jedoch zu einer Zunahme der Nekroptose und der Ausschüttung proinflammatorischer Zytokine, was darauf hindeutet, dass DAPK1 beim Lichen planus dem entzündlichen Zelltod und der Inflammation entgegenwirkt.^17^ Laufende Studien unserer Arbeitsgruppe befassen sich mit der Rolle weiterer Zelltodmechanismen wie Ferroptose und Pyroptose. Therapeutisch ergeben sich auf Basis dieser Studien zwei Möglichkeiten die Inflammation bei Typ‐1‐dominanten Hauterkrankungen zu kontrollieren. Einerseits durch die Blockade des IFN‐γ‐Signals und andererseits durch die Blockade des entzündlichen Zelltodes. Ersteres wurde bereits mehrfach beschrieben, da durch den Einsatz von Januskinase‐Inhibitoren (JAKi) die Möglichkeit besteht, die intrazelluläre Weiterleitung des INF‐γ‐Signals zu unterbinden. Zahlreiche Fallberichte beschreiben positive Effekte der JAK‐Inhibition, zum Beispiel beim Lichen planus oder beim kutanen Lupus erythematodes.^18^ Zulassungen von JAK‐Inhibitoren für die Therapie der Alopecia areata und der Vitiligo verdeutlichen das große Potenzial dieses therapeutischen Ansatzes.^19^, ^20^ Kürzlich konnten wir zeigen, dass die Inhibition der Januskinase TYK2 einen vielversprechenden Ansatz beim kutanen Lupus erythematodes darstellt, da sowohl in vitro als auch *ex vivo* kultivierte Hautbiopsien sowie eine Fallserie von Patienten mit verschiedenen Subformen des kutanen Lupus erythematodes ausgeprägte antiinflammatorische Effekte zeigten.^21^ Aktuell laufen mehrere klinische Studien, die den Einsatz von JAKi bei Lichen planus oder kutanem Lupus erythematodes untersuchen. Im Gegensatz dazu gibt es bisher keine klinischen Berichte über den Einsatz von Nekroptose‐Inhibitoren bei entzündlichen Hautkrankheiten. Dies ist vor allem darin begründet, dass es bisher keine zugelassenen Präparate gibt, welche für einen Off‐Label‐Einsatz zur Verfügung stehen. Insofern begründet sich das Konzept, entzündlichen Zelltod zur Therapie von Typ‐1‐dominanten Hautkrankheiten zu hemmen, bisher auf Laborstudien und mechanistische Überlegungen. So wurde kürzlich gezeigt, dass der UVB‐vermittelte Zelltod von Keratinozyten in vitro erfolgreich durch den RIPK3‐Inhibitor GSK’872 verhindert werden kann.^22^ Der Vorteil einer pharmakologischen Inhibition der Nekroptose beim Menschen wäre, dass durch diesen Ansatz im Gegensatz zur JAK‐Inhibition keine breite immunsuppressive Wirkung erzielt werden würde, da entzündlicher Zelltod nur im Bereich der Inflammation stattfindet. Mögliche Nachteile sind jedoch die unklare Verträglichkeit, insbesondere in Bezug auf virale Infektionen und Tumorerkrankungen, da entsprechend der natürlichen Funktion der Nekroptose, hier möglicherweise unerwünschte Effekte erzielt werden könnten. Die bisherigen Daten über den klinischen Einsatz von Nekroptose‐Inhibitoren bei anderen Erkrankungen zeigten keine negativen Sicherheitssignale, jedoch handelte es sich ausschließlich um Phase‐1‐ oder Phase‐2‐Studien mit begrenzter Population und Anwendungsdauer.^14^ Insofern bedarf es weiterer klinischer Daten, um das therapeutische Potenzial von neuen Molekülen, die den entzündlichen Zelltod blockieren, bei Typ‐1‐dominanten Hauterkrankungen besser abschätzen zu können.

## FAZIT FÜR DIE PRAXIS

Unsere Daten verdeutlichen, dass neben der Apoptose auch entzündlicher Zelltod bei der Interface‐Dermatitis aktiviert wird. Dadurch, dass dieser die lokale Entzündungsreaktion weiter verstärken kann, stellen die Schlüsselkinasen, die zum Beispiel die Nekroptose vermitteln, potenziell neue therapeutische Targets dar. Während in dermatologischen Indikationen bisher noch keine Nekroptose‐Inhibitoren getestet wurden, legen die Daten aus anderen Indikationen nahe, dass die pharmakologische Inhibition der Nekroptose grundsätzlich möglich und bei der bisher begrenzten Studienpopulation auch sicher ist. Insofern sind weitere Studien, vor allem klinische Wirksamkeits‐ und Sicherheitsstudien von Nekroptose Inhibitoren in der Dermatologie wünschenswert, um den hohen klinischen Bedarf für neue Therapien jenseits der JAKi für diese Krankheitsgruppe in Zukunft besser zu decken.

## FINANZIERUNG

Die Arbeit wurde durch das Advanced Clinician Scientist Program der Deutschen Stiftung Dermatologie e.V. in Zusammenarbeit mit der Deutschen Dermatologischen Gesellschaft e.V. (DDG; [https://derma.de/stipendien‐forschungspreise]) und der Arbeitsgemeinschaft Dermatologische Forschung e.V. (ADF; [https://www.adf‐online.de]) gefördert. Das Advanced Clinician Scientist Program der DDG wurde freundlich unterstützt durch Abbvie Deutschland, Almirall, Bristol Myers Squibb, Lilly, Novartis, Sanofi und UCB Pharma.

## CONFLICT OF INTEREST STATEMENT

F.L. war als Berater tätig und/oder erhielt Vortragshonorare von AbbVie, Almirall, Amgen, Biogen, Boehringer Ingelheim, Bristol Myers Squibb, Janssen, Johnson & Johnson, LEO Pharma, Lilly, Novartis, Roche, Sanofi, Regeneron, UCB, Union Therapeutics, Hexal und Incyte.
